# Fixed-Time Observer Based Prescribed-Time Containment Control of Unmanned Underwater Vehicles with Faults and Uncertainties

**DOI:** 10.3390/s19204515

**Published:** 2019-10-17

**Authors:** Tingting Yang, Shuanghe Yu

**Affiliations:** Department of Marine Electrical Engineering, Dalian Maritime University, Dalian 116026, China; tt.yang@dlmu.edu.cn

**Keywords:** containment control, UUVs, prescribed-time control, fixed-time observer

## Abstract

The problem of prescribed-time containment control of unmanned underwater vehicles (UUVs) with faults and uncertainties is considered. Different from both regular finite-time control and fixed-time control, the proposed prescribed-time control strategy is built upon a novel coordinate transformation function and the block decomposition technique, resulting in the followers being able to move into the convex hull spanned by the leaders in prespecifiable convergence time. Moreover, intermediate variables and the control input terms are also shown to remain uniformly bounded at the prescribed-time. To reduce the magnitude of the bounds, a novel fixed-time observer for the fault is proposed. Two numerical examples are provided to verify the effectiveness of the proposed prescribed-time control strategy.

## 1. Introduction

Formation control, a typical behavior in various aspects of systems, has received considerable attention due to its wide applications in spacecraft formation flying, deep-sea inspections, mobile robots and underwater vehicles. Many of the typical systems, the unmanned underwater vehicles (UUVs), share information with neighbors to obtain the goal in the complex ocean environment. In particular, containment control with multiple leaders is of great vital and potential application. For instance, the leaders can detect the obstacles, and the followers maintaining in the convex hull formed by the leaders can execute the task with collision avoidance [1,2,3,4].

In leader–follower formation control of UUVs, some challenging issues exist that deserve discussion, e.g., the convergence speed of the formation control system. In [5], the finite-time formation control of multiple nonholonomic mobile robots is considered. In [6], a finite-time leader–follower formation control for quadrotor aircraft is discussed, and a similar finite-time fault-tolerant leader–follower formation control strategy is presented for a group of autonomous surface vessels in [7]. In [8], the finite-time consensus and collision avoidance control algorithms for multiple UUVs are considered. Furthermore, in [9], fixed-time leader–follower formation control of autonomous underwater vehicles with event-triggered intermittent communications is presented, and the fixed-time formation control algorithm can not only ensure the settling time regardless of the initial conditions of the system, but also can obtain higher accuracy performance and faster convergence speed of the system. While fixed-time stabilization fixes the defects of the finite-time control algorithm, where the convergence time is set by some fixed number independently of the initial condition, it should be emphasized that the settling time in fixed-time control cannot be preassigned arbitrarily, due to the fact that the upper bound of settling time is subject to certain restrictions. Furthermore, the existing algorithms for finite-time control and fixed-time control do not always lead to smooth control action because of the existence of the signum function. In [10], the prescribed-time consensus is considered in the single integrator model. In [11], the prescribed finite-time consensus tracking for multi-agent systems with nonholonomic chained-form dynamics is considered. To the authors’ knowledge, few works related to the formation control for UUVs by smooth control law within fixed-time have been considered.

On the other hand, although finite-time and fixed-time stabilization have been widely considered due to the specified time property for the control system [12], most of the finite-time and fixed-time stabilization algorithms of a chain of integrators are presented by the approaches based on sliding modes and the concepts of homogeneity [13,14,15,16]. However, the tuning of control parameters is complicated, and the issue of high control gain always exists.

Motivated by the prescribed-time observer design in [17], the prescribed-time state feedback controller design [18] and the prescribed-time output feedback for linear systems in controllable canonical form in [19], in this paper, the containment control of multiple UUVs with faults and uncertainties in prescribed-time is investigated. By employing the consensus variables, the consensus problem is transformed into the stabilization of general MIMO systems. Due to the MIMO structure of the considered system, the original multi-input system needs to be decomposed into the block form [20]. However, due to the dimensions of the block subsystems being distinct, which increases the difficulty for the prescribed-time controller design, the stabilization algorithms for a chain of integrators lose efficacy and cannot be utilized directly. Thus, we propose a novel prescribed-time state feedback controller for MIMO linear systems by employing a novel nonsingular coordinate transformation function based on the block decomposition technique, which allows for both easy prescriptions of the convergence times, and minimal tuning of the observer and controller parameters. In addition, the bounds of the intermediate variables and the control inputs are obtained.

Compared with previous works [5,6,7,8,9,10,17,18,19], the contribution of this paper is at least threefold. First, in contrast to [5,6,7,8,9], whose converge time is related to the initial values or cannot be preassigned arbitrarily, the results obtained in this paper are the containment control scheme of multiple UUVs in prescribed-time, which can be arbitrarily assigned regardless of the system restrictions or the initial values. Moreover, the control law continuously avoids the signum function. Second, compared with [10,17,18], where the system is SISO, in this paper the MIMO case is solved. Since the block subsystems have distinct dimensions, the methods for the traditional chain system of the intermediate variable are inapplicable. Hence the existing prescribed-time control laws cannot be used here. To this end, different from [18] and [19], a novel intermediate variable dynamic system is introduced. On this basis, by the induction method, the non-singular coordinate transformation for the distinct dimension problem is proposed. Additionally, to confirm the relation between the UUV system and the transformed one, a special inverse transformation analytic solution is used. It is proven that the containment errors converge to zero, and the intermediate variables and the control input terms are uniformly bounded in the prescribed time, which increase the difficulties and challenges. Moreover, to reduce the magnitude of the bounds, a novel fixed-time observer of the fault is proposed. Third, compared with the recent literature [19], the containment control system is limited to the simple single Integrator system. The containment controllers proposed in this paper can be implemented in the multi-agent UUV systems, which is more practical and meaningful.

Notations: In this paper, $x^{T}$ represents the transpose of *x*. The vector $1_{N}$ is defined as ${\left[1,1,\ldots ,1\right]}^{T}\in R^{N}$. i∈I[1,N] means $i={\left[1,\cdots ,N\right]}^{T}$. Matrix $I_{N}$ is the N-dimensional identity matrix. ∥·∥ is represented as the Euclidean norm and ⊗ is the Kronecker product. $R^{m\times n}$ is the set of m×n real matrices.

## 2. Preliminaries and Problem Formulation

### 2.1. Preliminaries

Using graph theory, we can model the topology in a system consisting of *N* agents. Denoted by ∀i∈Γ the vertex set. Let g={Δ,E,A} be a directed graph of *N* orders, where $\Delta =\{v_{1},v_{2},\cdots ,v_{N}\}$ is a finite nonempty set of nodes, and E⊆Δ×Δ is the set of edges. The weighted adjacency matrix $A=\left[a_{ij}\right]\in R^{n\times n}$ is defined such that $a_{ij}$ is positive if $(v_{i},v_{j})\in E$, while $a_{ij}=0$ otherwise. If $(v_{j},v_{i})\in E$ is also satisfied, then the graph is undirected. The Laplacian matrix $L={\left[l_{ij}\right]}_{N\times N}$ is defined as $l_{ii}=\sum_{j=1,j\neq i}^{N}a_{ij}$ and $l_{ij}=-a_{ij},i\neq j,\;\forall i,j\in 1_{N}$. Both the adjacency matrix *A* and Laplacian matrix *L* are symmetric for undirected graphs. A directed graph contains a directed spinning tree if there exists a directed path from the root to every other node in the graph.

**Definition** **1** ([21]). *Given a set $\Omega \in R^{m},\;\forall x\in \Omega ,\;y\in \Omega ,\;0\leq \gamma \leq 1$, if (1−γ)x+γy∈Ω, then* Ω *is convex. For a finite set of points $y_{1},y_{2},\cdots ,y_{n}\in R^{m}$, the convex hull is*

(1) $$\begin{array}{c} co\left\{y_{1},y_{2},\ldots y_{n}\right\}=\left\{\sum_{i=1}^{n}\delta_{i}y_{i}\left|\delta_{i}\in R,\delta_{i}\geq 0,\right.\sum_{i=1}^{n}\delta_{i}=1\right\} \end{array}$$

*Consider M(M<N) followers labeled as 1 to M and N−M leaders labeled as N−M+1 to N. Define L as the Laplacian matrix of its corresponding topology. The matrix L can be described as*

(2) $$\begin{array}{c} L=\left[\begin{array}{cc} L_{1} & L_{2}\\ 0_{(N-M)\times M} & 0_{\left(N-M)\times (N-M\right)} \end{array}\right] \end{array}$$

*where $L_{1}=R^{M\times M}$ and $L_{2}\in R^{M\times (N-M)}$.*

**Definition** **2** ([21]). *The containment control is achieved when the followers converge to the convex hull formed by the leaders. That is to say, when t→∞, $x_{i}\left(t\right)\to co\left\{x_{j}\left(t\right)\left|i\right.\in le\right\},i\in F$.*

**Lemma** **1** ([22]). *In the directed graph, the matrix $L_{1}\in R^{M\times N}$ is invertible if it contains a directed spanning tree.*

**Lemma** **2** ([23]). *In a directed graph, if there is a directed spanning tree, the sum of the elements in each row of the matrix $L_{1}^{-1}L_{2}$ is 1.*

### 2.2. Problem Formulation

Consider a network of a multi-agent UUV system consisting of *M* followers, labeled as UUV 1 to *M*. The nonlinear maneuvering model of the UUV can be described below [Fossen, 2002]:

(3) $$\begin{array}{c} \begin{array}{c} M_{i}{\dot{\nu }}_{i}+C\left(\nu_{i}\right)\nu_{i}+D\left(\nu_{i}\right)\nu_{i}=-g\left(\eta_{i}\right)+\tau_{i},\\ {\dot{\eta }}_{i}=J\left(\eta_{i}\right)\nu_{i}, \end{array} \end{array}$$

where $\eta_{i}={\left[n_{i},e_{i},\psi_{i}\right]}^{T}$ is the standard position vector in the inertial coordinate system, $\nu_{i}={\left[\varrho_{i},v_{i},\vartheta_{i}\right]}^{T}$ is the standard velocity vector in body coordinate system. $n_{i},e_{i}$ are, respectively, the position in north and east, $\varrho_{i},v_{i}$ are, respectively, the velocity in surge and sway. Moreover, the variables $\psi_{i}$ and $\vartheta_{i}$ are the angles and rates in yaw, respectively. Define $p_{i}={\left[n_{i},w_{i}\right]}^{T}$ and $\epsilon_{i}={\left[\varrho_{i},v_{i}\right]}^{T}$. The control input vector $\tau_{i}={\left[\tau_{xi},\tau_{yi},\tau_{\psi i}\right]}^{T}$ is composed of surge force $\tau_{xi}$, sway force $\tau_{yi}$ and yaw moment $\tau_{\psi i}$. The matrix $C(\nu_{i})$ represents rigid-body Coriolis-centripetal matrix and $D(\nu_{i})$ is the damping matrix. $g(\eta_{i})$ is the matrix of restoring forces. $J(\eta_{i})$ denotes the kinematic transformation matrix from the body-fixed reference frame to the inertial frame. They are assumed to be known matrices of compatible dimensions. Moreover,

$$M_{i}=diag\left\{m-X_{{\dot{\varrho }}_{i}},m-Y_{{\dot{v}}_{i}},I_{z}\right\}$$

$$C\left(\nu_{i}\right)=\left[\begin{array}{ccc} 0 & 0 & -mv_{i}+Y_{{\dot{v}}_{i}}v_{i}\\ 0 & 0 & m\rho_{i}-X_{{\dot{\varrho }}_{i}}\varrho_{i}\\ mv_{i}-Y_{{\dot{v}}_{i}}v_{i} & -m\varrho_{i}+X_{{\dot{\varrho }}_{i}}\varrho_{i} & 0 \end{array}\right]$$

$$D\left(\nu_{i}\right)=diag\{-X_{\varrho_{i}}-X_{\varrho_{i}\left|\varrho_{i}\right|}|\varrho_{i}|,-Y_{v_{i}}-Y_{v_{i}\left|v_{i}\right|}|v_{i}|,-N_{\vartheta_{i}}-N_{\vartheta_{i}\left|\vartheta_{i}\right|}|\vartheta_{i}\left|\right\}.$$

where $X_{{\dot{\varrho }}_{i}}$ and $Y_{{\dot{v}}_{i}}$ are added mass terms.

**Lemma** **3.** *Define $x_{fi}={\left[p_{i}^{T},\epsilon_{i}^{T}\right]}^{T}$ as the position and velocity of the i-th UUV, then the nonlinear UUV maneuvering System (3) can be equivalent to the dynamics below,*

(4) $$\begin{array}{c} \begin{array}{c} {\dot{x}}_{fi}=A_{i}x_{fi}+B\tau_{i}^{\prime },\\ y_{fi}=Cx_{fi}, \end{array} \end{array}$$

(5) $$\begin{array}{c} \begin{array}{c} {\dot{\psi }}_{i}=\vartheta_{i}\\ {\dot{\vartheta }}_{i}=-\frac{d_{33}}{m_{33}}\vartheta_{i}+\frac{1}{m_{33}}\tau_{\psi_{i}} \end{array} \end{array}$$

*with*

$$A_{i}=\left[\begin{array}{cc} 0 & I\\ 0 & A_{\lambda_{i}} \end{array}\right],A_{\lambda_{i}}=diag\left\{\frac{d_{11}}{m_{11}},\frac{d_{22}}{m_{22}}\right\},B=\left[\begin{array}{c} 0\\ I \end{array}\right],C=\left[\begin{array}{cc} I & 0 \end{array}\right],\tau_{i}^{\prime }=R\left(\psi_{i}\right)M_{1}^{-1}\tau_{i}=\left[\tau_{i1}^{\prime };\tau_{i2}^{\prime }\right],$$

$$R\left(\psi_{i}\right)=\left[\begin{array}{cc} cos(\psi_{i}) & -sin(\psi_{i})\\ sin(\psi_{i}) & cos(\psi_{i}) \end{array}\right],\tau_{i}=\left[\begin{array}{c} \tau_{xi}\\ \tau_{yi} \end{array}\right].$$

**Proof.**  See Appendix A. □

Due to the complex ocean environment, UUVs are inevitably affected by uncertainties or suffer from faults. Hence, this paper solves the containment control of multi-agent UUV systems with faults and uncertainties. Then the dynamics of Equation (4) can be extended as follows,

(6) $$\begin{array}{c} \begin{array}{c} {\dot{x}}_{fi}=Ax_{fi}+B\tau_{i}^{\prime }+F_{a}f_{i}+Dd_{i},\\ y_{fi}=Cx_{fi},\;\;i\in F \end{array} \end{array}$$

where the symbol *F* represents the set of followers, $x_{fi}\in R^{n}$ is the state of the *i*-th UUV, $\tau_{i}^{\prime }\in R^{m}$ and $y_{fi}\in R^{r}$ are, respectively, the input and output state, $\;d_{i}\in R^{q}$ represents the disturbances on sensors and inputs, $f_{ai}\in R^{m}$ is the actuator faults. Moreover, we assume that the disturbances $d_{i}$ and faults $f_{ai}$ are matched, e.g., $F_{a}=BF_{a}^{\prime }$ and $D=BD^{\prime }$ where $F_{a}^{\prime }$ and $D^{\prime }$ have appropriate dimensions.

Consider the dynamics of the virtual leader UUV as follows,

(7) $$\begin{array}{c} \begin{array}{c} {\dot{x}}_{ri}=Ax_{ri},\\ y_{ri}=Cx_{ri},i\in le \end{array} \end{array}$$

where the symbol le represents the set of leaders, $x_{ri}\left(t\right)\in R^{n}$ is the state of leaders and $y_{ri}\left(t\right)\in R^{r}$ is the output of the leader.

### 2.3. Objective

This paper aims to design a prescribed-time containment control law for the multi-UUV System (3) under uncertainties and actuator faults, such that the trajectories of UUVs converge to the convex hull spanned by the leaders; i.e.,

(8) $$\begin{array}{c} \lim_{t\to T}dist\left\{x_{fi}\left(t\right),co\left\{x_{rj}\left(t\right)|i\in F,j\in le\right\}\right\}=0 \end{array}$$

where *T* is the prescribed time constant.

The main significance of the prescribed-time control lies in achieving the objective within the desired time without oscillations. For this, it is important for the multi-UUV system to perform some time-related tasks. Meanwhile, actuator faults have not been considered in the previous prescribed-time control research and make the problem more challenging.

In the following design, we first present a novel fixed-time observer to estimate the faults, which will reduce the magnitudes of the containment error variable and intermediate variables introduced in the prescribed-time control law. Next, to achieve the containment control for UUVs, a prescribed-time control law is proposed for a generalized MIMO system. Then, we employ the prescribed-time control method to develop the prescribed-time containment controllers for UUVs in Section 3.

## 3. Main Results

### 3.1. Model Transformation

Introduce the local neighborhood error variable below,

(9) $$\begin{array}{c} r_{i}=\sum_{j=1}^{M}a_{ij}\left(x_{i}-x_{j}\right)+\sum_{k=M+1}^{N}a_{ik}\left(x_{i}-x_{k}\right),i\in 1,\cdots ,M \end{array}$$

and the relative output information can be represented as

(10) $$\begin{array}{c} \xi_{i}=\sum_{j\in N_{i}}a_{ij}\left(y_{i}-y_{j}\right) \end{array}$$

According to Dynamic (6), by taking the derivative of the containment variable Dynamic (9), we have

(11) $$\begin{array}{c} \dot{r}=\left(L_{1}⊗I\right){\dot{x}}_{f}+\left(L_{2}⊗I\right){\dot{x}}_{r} \end{array}$$

Then it holds that

(12) $$\begin{array}{c} \begin{array}{c} \dot{r}=\left(I_{M}⊗A\right)r+\left(I_{M}⊗B\right)u+\left(I_{M}⊗B\right)\bar{f}+\left(I_{M}⊗D\right)\bar{d} \end{array} \end{array}$$

where $u=\left(L_{1}⊗I_{m}\right)\tau^{\prime }$, $\bar{f}=\left(L_{1}⊗I_{m}\right)f$, $\bar{d}=\left(L_{1}⊗I_{q}\right)d$. Thus, Dynamic (12) is equivalent to the subsystems below,

(13) $$\begin{array}{c} {\dot{r}}_{i}=Ar_{i}+Bu_{i}+F_{a}{\bar{f}}_{i}+D{\bar{d}}_{i} \end{array}$$

Combined with the relative output information of Equation (10), we get the following subsystems,

(14) $$\begin{array}{c} \begin{array}{c} {\dot{r}}_{i}=Ar_{i}+Bu_{i}+F_{a}{\bar{f}}_{i}+D{\bar{d}}_{i}\\ \xi_{i}=Cr_{i} \end{array} \end{array}$$

Then the prescribed-time containment control problem is transformed into the prescribed-time stabilization of Dynamic (14).

### 3.2. Fault Estimation

Define $\delta_{i}={\left[{\bar{r}}_{i}^{T},{\bar{f}}_{i}^{T}\right]}^{T}$, then Dynamic (14) can be written as

(15) $$\begin{array}{c} \begin{array}{c} \delta_{i}\left(t\right)=\bar{A}\delta_{i}\left(t\right)+\bar{B}u_{i}+\bar{D}{\bar{\beta }}_{i}\\ {\bar{\xi }}_{i}\left(t\right)=\bar{C}\delta_{i}\left(t\right), \end{array} \end{array}$$

with $\bar{A}=\left[\begin{array}{cc} A & F\\ 0 & 0 \end{array}\right]$, $\delta_{i}=\left[\begin{array}{c} {\bar{r}}_{i}\\ {\bar{f}}_{i} \end{array}\right]$, $\bar{B}=\left[\begin{array}{c} B\\ 0 \end{array}\right]$, ${\bar{\beta }}_{i}=\left[\begin{array}{c} {\bar{d}}_{i}\\ {\dot{\bar{f}}}_{i} \end{array}\right]$, $\bar{D}=\left[\begin{array}{cc} D & 0\\ 0 & I \end{array}\right]$, $\bar{C}=\left[\begin{array}{cc} C & 0 \end{array}\right]$. Design the fixed time fault estimator below,

(16) $$\begin{array}{c} \begin{array}{c} {\dot{z}}_{i}=M_{i1}z_{i}+G_{i}u_{i}+R_{i}\xi_{i}\\ {\hat{\bar{f}}}_{i1}=z_{i}+H_{i}\xi_{i} \end{array} \end{array}$$

(17) $$\begin{array}{c} \begin{array}{c} {\dot{z}}_{i}=M_{i2}z_{i}+G_{i}u_{i}+R_{i}\xi_{i}\\ {\hat{\bar{f}}}_{i2}=z_{i}+H_{i}\xi_{i} \end{array} \end{array}$$

**Theorem** **1.** *Consider the Dynamic (14) and the Observers (16) and (17), the fault estimation error dynamic is fixed-time bounded. Define $\rho_{i}=\left[\begin{array}{c} {\hat{\bar{f}}}_{i1}\\ {\hat{\bar{f}}}_{i2} \end{array}\right]$, then with t>τ,τ>0 the fault can be approximately estimated as ${\hat{\bar{f}}}_{i}=K_{i}\left(\rho_{i}\left(t\right)-e^{{\bar{M}}_{i}\tau }\rho_{i}\left(t-\tau \right)\right)$, $K_{i}=\left[I\;\;0\right]{\left[\begin{array}{cc} I & e^{{\bar{M}}_{i1}\tau }\\ I & e^{{\bar{M}}_{i2}\tau } \end{array}\right]}^{-1}$, ${\bar{M}}_{i}=\left[\begin{array}{cc} M_{i1} & 0\\ 0 & M_{i2} \end{array}\right]$, ${\bar{G}}_{i}=\left[\begin{array}{c} G_{i}\\ G_{i} \end{array}\right]$, ${\bar{R}}_{i}=\left[\begin{array}{c} R_{i}\\ R_{i} \end{array}\right]$, while satisfying the following matrix matching equations*

(18) $$\begin{array}{c} \begin{array}{c} M_{i}T_{i}+R_{i}\bar{C}-T_{i}\bar{A}=0\\ G_{i}-T_{i}B=0\\ T_{i}=L^{\prime }-H_{i}\bar{C}\\ M_{i1}\;\;\mathrm{and}\;\;M_{i2}\;\;\mathrm{are}\;\;\;\mathrm{Hurwitz}. \end{array} \end{array}$$

*with $L^{\prime }=\left[0\;I\right]$, $T_{i}=L^{\prime }-H_{i}\bar{C}$.*

**Proof.**  See Appendix B. □

**Remark** **1.** *The fixed-time observer design for the existing faults are necessary, which reduces the magnitude of intermediate variables and the control input. More details will be discussed in the next section.*

### 3.3. Prescribed Time Consensus Controller Design

In this section, the prescribed-time containment controller for multiple UUVs is considered. In fact, due to the system dynamics structure of Dynamic (14), the prescribed-time control of Dynamic (14) can be transformed to stabilized the generalized linear model in prescribed-time below,

(19) $$\begin{array}{c} \dot{x}\left(t\right)=Ax\left(t\right)+Bu\left(t\right)+\delta \left(t,x\left(t\right)\right) \end{array}$$

where $x\in R^{n}$ is the state vector, $u\in R^{m}$ is the control input. The term δ(t,x(t))=Bγ(t,x(t)) represents the matched faults and uncertainties. It is clear that the matrix pair (A,B) is controllable.

To sum up, the prescribed-time state for linear systems in the controllable canonical form is investigated [10,17,18]; however, due to the MIMO structure of the considered system, the original multi-input system needs to be decomposed into the block form, see [20]. However, due to the dimensions of the block subsystems being distinct, which increases the difficulty for the controller design, the prescribed-time stabilization algorithms of a chain of integrators lose efficacy and cannot be utilized directly. To further illustrate the prescribed-time containment control for UUVs, some results need to be given in advance.

#### 3.3.1. Block Decomposition

Let us initially decompose the original multi-input System (19) to a block form [24]. Below we use the known block decomposition procedure discussed in [25,26]. Let the orthogonal matrices $T_{i}$ be defined by the following algorithm: Initialization: $A_{0}=A$, $B_{0}=B$, $T_{0}=I_{n}$, *k* = 1. While rank $\left(B_{k}\right)=$ rown $\left(A_{k}\right)\;$ do $A_{k+1}=B_{k}^{⊥}A_{k}{\left(B_{k}^{⊥}\right)}^{T}$, $B_{k+1}=B_{k}^{⊥}A_{k}{\hat{B}}_{k}$, $T_{k+1}=\left(\begin{array}{c} B_{k}^{⊥}\\ {\hat{B}}_{k} \end{array}\right)$, k=k+1 and $B_{k}^{⊥}={\left(\mathrm{null}\left(B_{k}^{T}\right)\right)}^{T}\;$, ${\hat{B}}_{k}={\left(\mathrm{null}\left(B_{k}^{⊥}\right)\right)}^{T}$.

Then the orthogonal matrix *G* is obtained

(20) $$\begin{array}{c} G=\left(\begin{array}{cc} T_{k} & 0\\ 0 & I_{\mathrm{wk}} \end{array}\right)\left(\begin{array}{cc} T_{k-1} & 0\\ 0 & I_{wk-1} \end{array}\right)\cdots \left(\begin{array}{cc} T_{2} & 0\\ 0 & I_{w2} \end{array}\right)T_{1} \end{array}$$

with $w_{i}=n-rown\left(T_{i}\right)$ and

(21) $$\begin{array}{c} GAG=\left[\begin{array}{ccccc} A_{1,1} & A_{1,2} & 0 & \cdots & 0\\ A_{2,1} & A_{2,2} & A_{2,3} & \cdots & 0\\ \cdots & \cdots & \cdots & \cdots & \cdots \\ A_{k-1,1} & A_{k-1,2} & \cdots & A_{k-1,k-1} & A_{k-1,k}\\ A_{k,1} & A_{k,2} & \cdots & A_{k,k-1} & A_{k,k} \end{array}\right] \end{array}$$

$$GB={\left(0\;\;\;0\;\cdots \;\;0\;\;A_{k,k+1}^{T}\right)}^{T}$$

with $A_{k,k+1}={\hat{B}}_{0}B_{0}$, $A_{\mathrm{ij}}\in R^{n_{i}\times n_{j}}$, $n_{i}=rank\left(B_{k-i}\right)$, i,j=1,2,⋯,k and rank $\left(A_{i,i+1}\right)=n_{i}$.

It is clearly noted that the MIMO structure of System (19) is the specific one where k=0. Since rank $A_{i,i+1}=n_{i}=rown\left(A_{i,i+1}\right)$, then $A_{k,\;k+1}$ is invertible, and $A_{i,i+1}^{+}=A_{i,\;i+1}^{T}{\left(A_{i,\;i+1}A_{i,\;i+1}^{T}\right)}^{-1}$ is the right inverse matrix of $A_{i,\;i+1}$. Introduce the linear coordinate transformation s=Φy, $s={\left(s_{1},\cdots ,s_{k}\right)}^{T}$, $s_{i}\in R^{n_{i}}$, $y={\left(y_{i},\cdots ,y_{k}\right)}^{T}$, $y_{i}\in R^{n_{i}}$ by the formulas:

(22) $$\begin{array}{c} s_{i}=y_{i}+\phi_{i},\;i=1,\cdots ,k \end{array}$$

(23) $$\begin{array}{c} \phi_{i}=0,\;\phi_{i+1}=A_{i,i+1}^{+}\left(\sum_{j=1}^{i}A_{\mathrm{ij}}y_{j}+\sum_{r=1}^{i}\frac{\partial \phi_{i}}{\partial y_{r}}\sum_{j=1}^{r+1}A_{\mathrm{rj}}y_{j}\right) \end{array}$$

The presented coordinate transformation is linear and nonsingular. The inverse transformation $y=\phi^{-1}s$ is defined as follows:

(24) $$\begin{array}{c} y_{i}=s_{i}+\psi_{i},\;i=1,\cdots ,k \end{array}$$

(25) $$\begin{array}{c} \psi_{1}=0,\;\psi_{i+1}=A_{i,i+1}^{+}\left(\sum_{k=1}^{i}\frac{\partial \psi_{i}}{\partial s_{k}}A_{i,i+1}s_{k+1}+\sum_{j=1}^{i}A_{i,j}\left(s_{j}+\psi_{j}\right)\right) \end{array}$$

Then applying the transformation s=ΦGx, one has

(26) $$\begin{array}{c} \dot{s}=\left[\begin{array}{ccccc} 0 & A_{1,2} & 0 & \cdots & 0\\ 0 & 0 & A_{2,3} & \cdots & 0\\ \cdots & \cdots & \cdots & \cdots & \cdots \\ 0 & 0 & 0 & 0 & A_{k-1,k}\\ {\tilde{A}}_{k,1} & {\tilde{A}}_{k,2} & \cdots & {\tilde{A}}_{k,k-1} & {\tilde{A}}_{k,k} \end{array}\right]s+B^{{}^{\prime }}\left(u+\gamma \left(t,s\right)\right) \end{array}$$

with $B^{{}^{\prime }}=\Phi GB={\left(0\;\;0\cdots 0\;A_{k,k+1}^{T}\right)}^{T}$.

Next, we will propose a novel prescribed-time state feedback controller for MIMO linear systems by employing a novel nonsingular coordinate transformation function based on the block decomposition technique, which allows for both easy prescriptions of the convergence times, and minimal tuning of the observer and controller parameters. In addition, the bounds of the intermediate variables and the control inputs are obtained.

#### 3.3.2. Prescribed-Time Controller Design

To obtain the prescribed-time controller, both [17] and [18] introduce the scaling function as follows,

(27) $$\begin{array}{c} \mu_{1}\left(t-t_{0},T\right):=\frac{1}{T+t_{0}-t},\;t\in \left[t_{0},t_{0}+T\right] \end{array}$$

which is positive monotonic. When $t=t_{0},\;\mu_{1}=\frac{1}{T}$ and when $t=T+t_{0},\;\mu_{1}=1$. In addition, T>0 is freely prescribed by the user and independent of initial conditions. Following the above results, we propose the coordinate transformation w=P(s) by the following formulas:

**Lemma** **4.** *Consider the coordinate transformation w=P(s) for t∈[0,T) as follows,*

(28) $$\begin{array}{c} w_{i}=\frac{s_{i}}{T-t}+p_{i},\;\;p_{1}=0, \end{array}$$

(29) $$\begin{array}{c} p_{i+1}=\sum_{j=1}^{i}\frac{a_{i+1,j}}{{\left(T-t\right)}^{i+2-j}}s_{j},\;\;1\leq j\leq i\leq k \end{array}$$

*where the coefficients $a_{i,j}$ are a constant matrix to be determined as,*

(30) $$\begin{array}{c} a_{i,0}=0 \end{array}$$

(31) $$\begin{array}{c} a_{i,q}=0,\;q>i \end{array}$$

*and the recursion relations*

(32) $$\begin{array}{c} a_{i+1,j}=A_{i,i+1}^{+}\left(a_{i,j}\left(i+1-j+k_{1}\right)+a_{i,j-1}A_{i-1,j}\right). \end{array}$$

*Then it holds that*

(33) $$\begin{array}{c} {\dot{w}}_{i}=\frac{-k_{1}}{T-t}w_{i}+A_{i,i+1}w_{i+1},\;i=1,\cdots ,k-1 \end{array}$$

**Proof.**  See Appendix C. □

**Lemma** **5.** *The inverse coordinate transformation $s=P^{-1}\left(w\right)$ for t∈[0,T) can be described as follows,*

(34) $$\begin{array}{c} s_{i}=w_{i}\left(T-t\right)+l_{i},\;{\;l}_{1}=0, \end{array}$$

(35) $$\begin{array}{c} l_{i+1}=\sum_{j=1}^{i}b_{i,j}{\left(T-t\right)}^{1-i+j}w_{j},\;\;1\leq j\leq i\leq k \end{array}$$

*where the coefficients $b_{i,j}$ are constant matrix to be determined as,*

(36) $$\begin{array}{c} b_{i,0}=0 \end{array}$$

(37) $$\begin{array}{c} b_{i,q}=0,\;q>i \end{array}$$

*and the recursion relations*

(38) $$\begin{array}{c} b_{i+1,j}=A_{i,i+1}^{+}\left(-b_{i,j}\left(1-i+j+k_{1}\right)+b_{i,j-1}A_{j-1,j}\right). \end{array}$$

**Proof.**  See Appendix D. □

**Remark** **2.** *If k=3, then*

$$w=\left[\begin{array}{ccc} \frac{1}{T-t}I_{n1} & 0 & 0\\ \frac{A_{12}^{+}\left(k_{1}+1\right)}{{\left(T-t\right)}^{2}\;} & \frac{1}{T-t}I_{n2} & 0\\ \frac{{A_{23}^{+}A}_{12}^{+}\left(k_{1}+1\right)\left(k_{1}+2\right)}{{\left(T-t\right)}^{3}\;} & \frac{A_{23}^{+}\left(k_{1}+1\right)\left(A_{12}^{+}A_{12}+1\right)}{{\left(T-t\right)}^{2}\;} & \frac{1}{T-t}I_{n3} \end{array}\right]s$$

$$s=\left[\begin{array}{ccc} \left(T-t\right)I_{n1} & 0 & 0\\ -A_{12}^{+}\left(k_{1}+1\right) & \left(T-t\right)I_{n2} & 0\\ A_{23}^{+}A_{12}^{+}\left(k_{1}+1\right)k_{1}{\left(T-t\right)}^{-1} & -A_{23}^{+}\left(k_{1}+1\right)\left(A_{12}^{+}A_{12}+1\right) & \left(T-t\right)I_{n3} \end{array}\right]w$$

*where n1, n2, n3 are the dimensions of subsystems.*

**Remark** **3.** *Due to the dimensions of the block subsystems being distinct, the traditional chain system of the intermediate variable proposed in [18,19] is not applicative, and a novel intermediate variable dynamic system is introduced as Equation (28). To achieve the prescribed-time control, the novel coordinate transformation w=P(s) and the inverse coordinate transformation $s=P^{-1}\left(w\right)$ based on the block decomposition technique are introduced to deal with the difficulty caused by distinct dimensions.*

Applying the transformation w=P(s), we obtain the derivative of $w_{k}$,

(39) $$\begin{array}{ll} {\dot{w}}_{k} & =\frac{s_{k}}{{\left(T-t\right)}^{2}}+\frac{{\dot{s}}_{k}}{T-t}+\sum_{j=1}^{k-1}\frac{a_{k,j}\left(k+1-j\right)}{{\left(T-t\right)}^{k+2-j}}s_{j}+\sum_{j=1}^{k-1}\frac{a_{k,j}A_{j,\;j+1}}{{\left(T-t\right)}^{k+1-j}}s_{j+1}\\ & =\frac{s_{k}}{{\left(T-t\right)}^{2}}+\frac{A_{k,k+1}\left(\sum_{q=1}^{k}{\tilde{A}}_{k,q}s_{q}+u+\gamma \left(t,w\right)\right)}{T-t}\\ & +\sum_{j=1}^{k-1}\frac{a_{k,j}\left(k+1-j\right)}{{\left(T-t\right)}^{k+2-j}}s_{j}+\sum_{j=1}^{k-1}\frac{a_{k,j}A_{j,\;j+1}}{{\left(T-t\right)}^{k+1-j}}s_{j+1} \end{array}$$

Then the prescribed-time stabilization control for System (19) can be summarized as follows,

**Theorem** **2.** *Given the coordinate transformation in Lemmas 4 and 5, the block subsystems of dynamics w can be presented,*

(40) $$\begin{array}{c} \begin{array}{c} {\dot{w}}_{1}=-\frac{k_{1}}{T-t}w_{1}+A_{1,2}w_{2}\\ {\dot{w}}_{2}=-\frac{k_{1}}{T-t}w_{2}+A_{2,3}w_{3}\\ \vdots \\ {\dot{w}}_{k-1}=-\frac{k_{1}}{T-t}w_{k-1}+A_{k-1,k}w_{k}\\ {\dot{w}}_{k}=-\frac{k_{1}}{T-t}w_{k}+\frac{A_{k,k+1}}{T-t}\gamma \left(t,w\right) \end{array} \end{array}$$

*with the controller designed as*

(41) $$\begin{array}{c} \begin{array}{c} u=-\sum_{q=1}^{k}{\tilde{A}}_{k,q}s_{q}+\left(T-t\right)A_{k,k+1}^{+}(-\frac{s_{k}}{{\left(T-t\right)}^{2}}-\sum_{j=1}^{k-1}\frac{a_{k,j}\left(k+1-j\right)}{{\left(T-t\right)}^{k+2-j}}s_{j}\\ -\sum_{j=1}^{k-1}\frac{a_{k,j}A_{j,\;j+1}}{{\left(T-t\right)}^{k+1-j}}s_{j+1}-\frac{k_{1}}{T-t}w_{k}) \end{array} \end{array}$$

*Then the intermediate variable w and the control input are prescribed-time uniformly bounded, and the states of x and s are prescribed-time stabilized for t∈[0,T).*

**Proof.**  Denote $V_{i}=\frac{w_{i}^{T}w_{i}}{2},i=1,\ldots ,k$, whose derivative along the solution of Equation (40) is

(42) $$\begin{array}{c} {\dot{V}}_{k}=-\frac{k_{1}}{T-t}w_{k}^{T}w_{k}+\frac{1}{T-t}w_{k}^{T}A_{k,k+1}\gamma \left(t,w\right) \end{array}$$

By applying Young’s inequality with λ>0,

(43) $$\begin{array}{ll} {\dot{V}}_{k} & \leq -\frac{\left(k_{1}-\lambda \right){\left\|A_{k,k+1}\right\|}^{2}}{T-t}w_{k}^{T}w_{k}+\frac{1}{4\left(T-t\right)\lambda }\gamma^{2}\left(t,w\right)\\ & \leq -\frac{2\left(k_{1}-\lambda \right){\left\|A_{k,k+1}\right\|}^{2}}{T-t}V_{k}\left(t\right)+\frac{1}{4\left(T-t\right)\lambda }\gamma^{2}\left(t,w\right) \end{array}$$

Then

(44) $$\begin{array}{ll} V_{k} & \left(t\right)\leq \exp^{-2\left(k_{1}-\lambda \right){\left\|A_{k,k+1}\right\|}^{2}\int_{0}^{t}\frac{1}{\left(T-\tau \right)}d\tau }V\left(0\right)+\frac{1}{4\lambda }\int_{0}^{t}exp^{-2\left(k_{1}-\lambda \right)\int_{\tau }^{t}\frac{1}{\left((T-s\right)}ds}\gamma^{2}\left(\tau ,w\right)\frac{1}{T-\tau }d\tau \\ & \leq exp^{-2\left(k_{1}-\lambda \right){\left\|A_{k,k+1}\right\|}^{2}\left[ln\left(T\right)-ln\left(T-t\right)\right]}V\left(0\right)+\frac{{\left\|\gamma \left(t,w\right)\right\|}_{\left[0,t\right]}^{2}}{4\lambda }\int_{0}^{t}exp^{2\left(k_{1}-\lambda \right)\left(\int_{0}^{\tau }\frac{1}{T-s}ds-\int_{0}^{t}\frac{1}{T-s}ds\right)}\frac{1}{T-\tau }d\tau \\ & \leq exp^{-2\left(k_{1}-\lambda \right){\left\|A_{k,k+1}\right\|}^{2}\left[ln\left(T\right)-ln\left(T-t\right)\right]}V\left(0\right)\\ & +\frac{{\left\|\gamma \left(t,w\right)\right\|}_{\left[0,t\right]}^{2}}{4\lambda }exp^{-2\left(k_{1}-\lambda \right)\int_{0}^{t}\frac{1}{T-s}ds}\int_{0}^{t}exp^{2\left(k_{1}-\lambda \right)\int_{0}^{\tau }\frac{1}{T-s}ds}d\int_{0}^{\tau }\frac{1}{T-s}ds\\ & =\Delta_{1}\left(t\right)+\frac{\|\gamma \left(t,w\right){\|}_{\left[0,t\right]}^{2}}{4\lambda }\exp^{-2\left(k_{1}-\lambda \right)\int_{0}^{t}\frac{1}{\left(T-s\right)}\mathrm{ds}}\frac{1}{2(k_{1}-\lambda )}\exp^{2\left(k_{1}-\lambda \right)\int_{0}^{\tau }\frac{1}{\left(T-s\right)}\mathrm{ds}}{\left.\;\right|}_{0}^{t}\\ & =\Delta_{1}\left(t\right)+\frac{\|\gamma \left(t,w\right){\|}_{\left[0,t\right]}^{2}}{4\lambda }\exp^{-2\left(k_{1}-\lambda \right)\int_{0}^{t}\frac{1}{\left(T-s\right)}\mathrm{ds}}\frac{1}{2(k_{1}-\lambda )}{(exp}^{2\left(k_{1}-\lambda \right)\int_{0}^{t}\frac{1}{\left(T-s\right)}\mathrm{ds}}-1)\\ & =\Delta_{1}\left(t\right)+\frac{\|\gamma \left(t,w\right){\|}_{\left[0,t\right]}^{2}}{4\lambda }\frac{1}{2(k_{1}-\lambda )}{(1-exp}^{-2\left(k_{1}-\lambda \right)\int_{0}^{t}\frac{1}{\left(T-s\right)}\mathrm{ds}}) \end{array}$$

where $\Delta_{1}\left(t\right)=exp^{-2\left(k_{1}-\lambda \right){\left\|A_{k,k+1}\right\|}^{2}\left[lnT-ln\left(T-t\right)\right]}V\left(0\right)$, and if t=0, $\Delta_{1}\left(0\right)=1$ and t=T, $\Delta_{1}\left(T\right)=0$. The function $\Delta_{1}\left(t\right)$ monotonically decreases. Thus, $V_{k}\leq 1+\frac{\|\gamma \left(t,w\right){\|}_{\left[0,t\right]}^{2}}{8\lambda (k_{1}-\lambda )}$, and $\|w_{k}{\|}_{\infty }\leq \sqrt{2+\frac{\|\gamma \left(t,w\right){\|}_{\left[0,t\right]}^{2}\;}{4\lambda \left(k_{1}-\lambda \right)}}=\epsilon_{k}$. Under the condition, if $\gamma \left(t,w\right)\equiv 0$, then $\lim_{t\to T}V_{k}\left(t\right)=0.$ With ${\dot{w}}_{k-1}=-\frac{k_{1}}{T-t}w_{k-1}+A_{k-1,k}w_{k}$, define the Lyapunov function $V_{k-1}=\frac{w_{k-1}^{2}}{2}$, the derivative of $V_{k-1}$,

(45) $$\begin{array}{ll} {\dot{V}}_{k-1} & =w_{k-1}{\dot{w}}_{k-1}=-\frac{k_{1}}{T-t}w_{k-1}^{T}w_{k-1}+w_{k-1}^{T}A_{k-1,k}w_{k}\\ & \leq -\frac{k_{1}}{T-t}w_{k-1}^{T}w_{k-1}+{\left\|A_{k,k+1}\right\|}^{2}\frac{\lambda w_{k-1}^{T}w_{k-1}}{T-t}+\left(T-t\right)\frac{w_{k}^{T}w_{k}}{\lambda }\\ & \leq -\frac{k_{1}-\lambda {\left\|A_{k,k+1}\right\|}^{2}}{T-t}w_{k-1}^{T}w_{k-1}+\frac{{T\epsilon }_{k}^{2}}{\lambda } \end{array}$$

it holds that

(46) $$\begin{array}{c} {\dot{V}}_{k-1}\leq -\frac{{2k}_{1}-2\lambda {\left\|A_{k,k+1}\right\|}^{2}}{T-t}V_{k-1}+\frac{{T\epsilon }_{k}^{2}}{\lambda } \end{array}$$

(47) $$\begin{array}{c} {\dot{V}}_{k-1}+\frac{{2k}_{1}-2\lambda {\left\|A_{k,k+1}\right\|}^{2}}{T-t}V_{k-1}\leq \;\frac{{T\epsilon }_{k}^{2}}{\lambda } \end{array}$$

Define $k_{2}={2k}_{1}-2\lambda {\left\|A_{k,k+1}\right\|}^{2}$, $\epsilon_{k-1}=\frac{{T\epsilon }_{k}^{2}}{\lambda }.$ Then

(48) $$\begin{array}{c} \begin{array}{c} {\dot{V}}_{k-1}+\frac{k_{2}}{T-t}V_{k-1}\leq \epsilon_{k-1} \end{array} \end{array}$$

(49) $$\begin{array}{c} \begin{array}{c} \dot{\left(e^{\int_{0}^{t}\frac{k_{2}}{T-s}\mathrm{ds}}V_{k-1}\right)}\leq e^{\int_{0}^{t}\frac{k_{2}}{T-s}\mathrm{ds}}\epsilon_{k-1} \end{array} \end{array}$$

with the fact that

(50) $$\begin{array}{c} \begin{array}{c} e^{\int_{0}^{t}\frac{k_{2}}{T-s}\mathrm{ds}}=e^{k_{2}\left(-\ln\left(T-s\right){\left.\;\right|}_{0}^{t}\right)}={\left(\frac{T}{T-t}\right)}^{k_{2}} \end{array} \end{array}$$

one has

(51) $$\begin{array}{c} \begin{array}{c} {\left(\frac{T}{T-t}\right)}^{k_{2}}V_{k-1}\leq V_{k-1}\left(0\right)+\epsilon_{k-1}{\int_{0}^{t}\left(\frac{T}{T-s}\right)}^{k_{2}}\mathrm{ds} \end{array} \end{array}$$

Thus

(52) $$\begin{array}{ll} V_{k-1} & \leq {\left(\frac{T-t}{T}\right)}^{k_{2}}\left(V_{k-1}\left(0\right)+\frac{\epsilon_{k-1}T^{k_{2}}}{k_{2}-1}\left(\frac{1}{{\left(T-t\right)}^{k_{2}-1}}-\frac{1}{T^{k_{2}-1}}\right)\right)\\ & ={\left(\frac{T-t}{T}\right)}^{k_{2}}V_{k-1}\left(0\right)+\frac{\epsilon_{k-1}T^{k_{2}}}{k_{2}-1}\left(\frac{T-t}{T^{k_{2}}}-\frac{{\left(T-t\right)}^{k_{2}}}{T^{2k_{2}-1}}\right) \end{array}$$

when t=T, $V_{k-1}=0$, then $w_{k-1}=0.$ Similarly, for i=k−2,⋯,1, $w_{i}=0.$ Then the intermediate variable *w* is prescribed-time uniformly bounded. With s=ΦGx, $s=P^{-1}w$, Φ and *G* are nonsingular transformation; we can know that the states of *x* and *s* are prescribed-time stabilized.

Since

(53) $$\begin{array}{c} \begin{array}{c} {\dot{w}}_{i-1}=-\frac{k_{1}}{T-t}w_{i-1}+A_{i-1,i}w_{i} \end{array} \end{array}$$

then

(54) $$\begin{array}{c} \begin{array}{c} \dot{\left(e^{\int_{0}^{t}\frac{k_{1}}{T-s}\mathrm{ds}}w_{i-1}\right)}\leq e^{\int_{0}^{t}\frac{k_{1}}{T-s}\mathrm{ds}}A_{i-1,i}w_{i} \end{array} \end{array}$$

Since

(55) $$\begin{array}{c} \begin{array}{c} e^{\int_{0}^{t}\frac{k_{1}}{T-s}\mathrm{ds}}=e^{k_{1}\left(-\ln\left(T-s\right){\left.\;\right|}_{0}^{t}\right)}={\left(\frac{T}{T-t}\right)}^{k_{1}} \end{array} \end{array}$$

then

(56) $$\begin{array}{ll} {\left(\frac{T}{T-t}\right)}^{k_{1}}w_{i-1} & \leq w_{i-1}\left(0\right)+A_{i-1,i}w_{i}{\int_{0}^{t}\left(\frac{T}{T-s}\right)}^{k_{1}}\mathrm{ds}\\ & \leq w_{i-1}\left(0\right)+A_{i-1,i}w_{i}\frac{T^{k_{1}}}{k_{1}-1}\left(\frac{1}{{\left(T-t\right)}^{k_{1}-1}}-\frac{1}{T^{k_{1}-1}}\right) \end{array}$$

then one holds

(57) $$\begin{array}{ll} w_{i-1} & \leq w_{i-1}\left(0\right){\left(\frac{T-t}{T}\right)}^{k_{1}}+A_{i-1,i}w_{i}\frac{1}{k_{1}-1}\left(\left(T-t\right)-\frac{{\left(T-t\right)}^{k_{1}}}{T^{k_{1}-1}}\right)\\ & =w_{i-1}\left(0\right){\left(\frac{T-t}{T}\right)}^{k_{1}}+A_{i-1,i}w_{i}\frac{\left(T-t\right)}{k_{1}-1}-\frac{{A_{i-1,i}w_{i}\left(T-t\right)}^{k_{1}}}{T^{k_{1}-1}\left(k_{1}-1\right)} \end{array}$$

Since $\|w_{k}{\|}_{\infty }\leq \epsilon_{k}$, then

(58) $$\begin{array}{ll} w_{k-1} & \leq \left(T-t\right)\left(w_{k-1}\left(0\right)\frac{{\left(T-t\right)}^{k_{1}-1}}{T^{k_{1}}}+A_{k-1,k}w_{k}\frac{1}{k_{1}-1}\left(1-\frac{{\left(T-t\right)}^{k_{1}-1}}{T^{k_{1}-1}}\right)\right)\\ & =\left(T-t\right)D_{k-1} \end{array}$$

where $D_{k-1}$ is bounded. Then

(59) $$\begin{array}{ll} w_{k-2} & \leq w_{k-2}\left(0\right){\left(\frac{T-t}{T}\right)}^{k_{1}}+A_{k-2,k-1}w_{k-1}\frac{\left(T-t\right)}{k_{1}-1}-\frac{{A_{k-2,k-1}w_{k-1}\left(T-t\right)}^{k_{1}}}{T^{k_{1}-1}\left(k_{1}-1\right)}\\ & =w_{k-2}\left(0\right){\left(\frac{T-t}{T}\right)}^{k_{1}}+A_{k-2,k-1}D_{k-1}\frac{{\left(T-t\right)}^{2}}{k_{1}-1}-\frac{{A_{k-2,k-1}D_{k-1}\left(T-t\right)}^{k_{1}+1}}{T^{k_{1}-1}\left(k_{1}-1\right)}\\ & ={\left(T-t\right)}^{2}\left(w_{k-2}\left(0\right)\frac{{\left(T-t\right)}^{k_{1}-2}}{T^{k_{1}}}+A_{k-2,k-1}D_{k-1}\frac{1}{k_{1}-1}\left(1-\frac{{\left(T-t\right)}^{k_{1}-1}}{T^{k_{1}-1}}\right)\right)\\ & ={\left(T-t\right)}^{2}D_{k-2} \end{array}$$

Then we can have $w_{k-q}\leq {\left(T-t\right)}^{q}D_{k-q}$, q=1,⋯,k−1.

With the coordinate transformation $s=P^{-1}\left(w\right)$, then

(60) $$\begin{array}{c} \begin{array}{c} s_{j+1}=\sum_{q=1}^{j+1}b_{j+1,q}{\left(T-t\right)}^{-j+q}w_{q} \end{array} \end{array}$$

and the control input is written as

(61) $$\begin{array}{ll} u & =\left(T-t\right)(A_{k,k+1}^{+}\left(-\sum_{q=1}^{k}{\tilde{A}}_{k,q}s_{q}-\frac{s_{k}}{{\left(T-t\right)}^{2}}\right)\\ & -\sum_{j=1}^{k-1}\frac{a_{k,j}\left(k+1-j\right)}{{\left(T-t\right)}^{k+2-j}}s_{j}-\sum_{j=1}^{k-1}\frac{a_{k,j}A_{j,\;j+1}}{{\left(T-t\right)}^{k+1-j}}\sum_{q=1}^{j+1}b_{j+1,q}{\left(T-t\right)}^{-j+q}w_{q}-\frac{k_{1}}{T-t}w_{k})\\ & -{\left(T-t\right)A}_{k,k+1}^{+}\sum_{q=1}^{k}{\tilde{A}}_{k,q}s_{q}-\frac{s_{k}}{T-t}-\sum_{j=1}^{k-1}\frac{a_{k,j}\left(k+1-j\right)}{{\left(T-t\right)}^{k+1-j}}s_{j}-\sum_{j=1}^{k-1}\sum_{q=1}^{j+1}\frac{a_{k,j}A_{j,\;j+1}b_{j+1,q}}{{\left(T-t\right)}^{k-q}}w_{q}-K_{1}w_{k} \end{array}$$

According to the fact that $w_{k}=\frac{s_{k}}{T-t}+\sum_{j=1}^{k-1}\frac{a_{k,j}}{{\left(T-t\right)}^{k+1-j}}s_{j}$ is bounded, $-\frac{s_{k}}{T-t}-\sum_{j=1}^{k-1}\frac{a_{k,j}\left(k+1-j\right)}{{\left(T-t\right)}^{k+1-j}}s_{j}$ is bounded. Further, because $w_{k-q}\leq {\left(T-t\right)}^{q}D_{k-q}$, by the simple transformation, let k−q=l, then $w_{l}={\left(T-t\right)}^{k-l}D_{l}$, one can know that $\frac{w_{q}}{{\left(T-t\right)}^{k-q}}$ is bounded. Then the control input is bounded.

The proof is completed. □

Immediately, the prescribed-time containment controller for UUVs can be given as follows,

**Theorem** **3.** *Consider the i-th UUV System (4) with fixed-time fault Observers (16) and (17) and the controller as follows*

(62) $$\begin{array}{c} u_{i}=-\sum_{q=1}^{k}{\tilde{A}}_{k,q}s_{q}+\left(T-t\right)A_{k,k+1}^{+}(-\frac{s_{k}}{{\left(T-t\right)}^{2}}-\sum_{j=1}^{k-1}\frac{a_{k,j}\left(k+1-j\right)}{{\left(T-t\right)}^{k+2-j}}s_{j}\\ -\sum_{j=1}^{k-1}\frac{a_{k,j}A_{j,\;j+1}}{{\left(T-t\right)}^{k+1-j}}s_{j+1}-\frac{k_{1}}{T-t}w_{k})-F_{a}^{\prime }{\hat{\bar{f}}}_{i},\\ \tau_{\psi_{i}}=d_{33}/m_{33}\vartheta_{2}+\left(T-t\right)\left(-\frac{\vartheta_{2}}{{\left(T-t\right)}^{2}}-\frac{2a_{2,1}}{{\left(T-t\right)}^{3}}\vartheta_{1}-\frac{a_{2,1}}{{\left(T-t\right)}^{2}}\vartheta_{2}-\frac{k_{1}}{T-t}w_{2}^{\prime }\right) \end{array}$$

*where k=2, ${\tilde{A}}_{2,1}=0$, ${\tilde{A}}_{2,2}=A_{\lambda_{i}}$, $A_{12}=I_{2}$, $A_{11}=0$, $a_{21}=1+k_{1}$ with designed parameter $k_{1}$, w=P(s) and $w^{\prime }=P\left(\vartheta \right)$, then the containment control objective of Equation (1) is achieved in prescribed-time, e.g., the containment variable $r_{i}=0$. Moreover, the intermediate variable w and the control input u are prescribed-time uniformly bounded for t∈[0,T).*

**Proof.**  Due to the fact that $V_{k}\leq 1+\frac{\|\gamma \left(t,w\right){\|}_{\left[0,t\right]}^{2}}{8\lambda (k_{1}-\lambda )}$, the existence of the fixed-time observer ${\hat{f}}_{i}$ transforms the term $\|\gamma \left(t,w\right){\|}_{\left[0,t\right]}^{2}=f_{i}^{2}+d_{i}^{2}$ into the value relative to $\alpha_{i}^{2}+d_{i}^{2}$, which reduces the magnitude of bounds by choosing appropriately the initial values of the observers and the parameter λ. The remaining proof is similar to Theorem 1. Due to the limited space, the proving process is omitted here. □

## 4. Simulation

In this section, two examples are given to demonstrate the merits and effectiveness of the prescribed-time controller.

**Figure 1:**
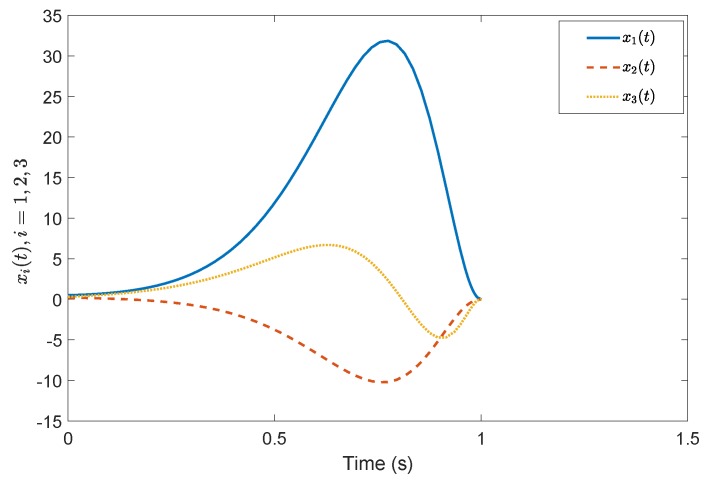
States *x*(t) by the prescribed-time controller.

**Figure 2:**
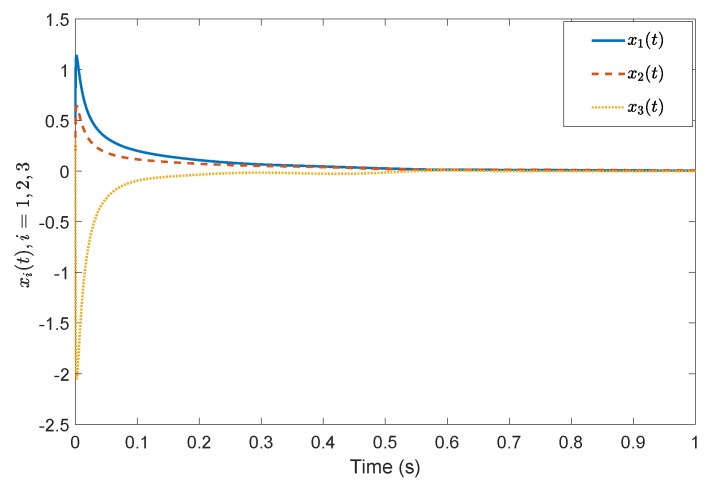
States *x*(t) by the fixed-time controller in [26].

**Figure 3:**
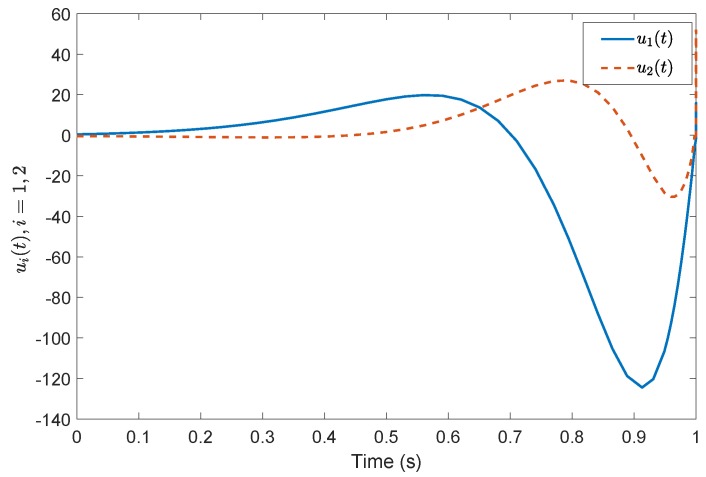
Control input *u*(t) by the prescribed-time controller.

**Figure 4:**
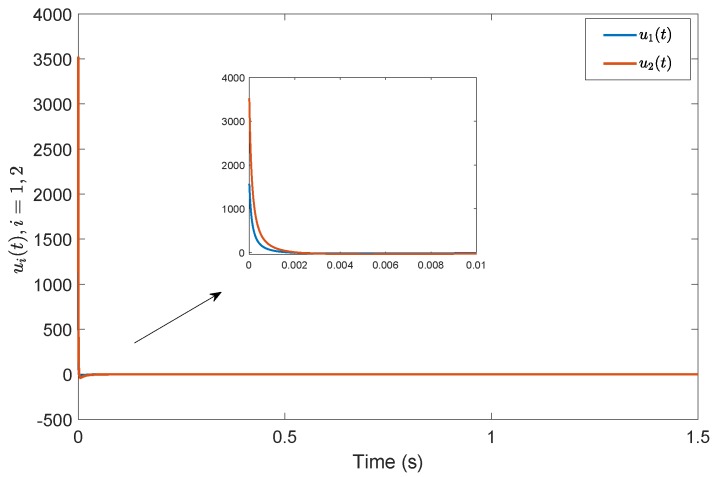
Control input *u*(t) by the fixed-time controller in [26].

**Example** **1.** *Consider a benchmark example, System (2), with $A=\left[\begin{array}{ccc} 1 & -3 & 2\\ -2 & 0 & 3\\ 0 & -1 & 4 \end{array}\right]$, $B=\left[\begin{array}{cc} 2 & 0\\ -1 & 1\\ 0 & -3 \end{array}\right]$, $G=\left[\begin{array}{ccc} 0.486 & 0.8571 & 0.2857\\ -0.8571 & 0.4857 & -0.1714\\ -0.2857 & -0.1714 & 0.9429 \end{array}\right]$, $A_{11}=-0.5918$, $A_{12}=\left[\begin{array}{cc} -0.4449 & 4.9469 \end{array}\right]$, $A_{21}=\left[\begin{array}{cc} 1.2980 & 0.7184 \end{array}\right]$, $A_{23}=\left[\begin{array}{cc} -2.200 & 1.0000\\ -0.4000 & -3.0000 \end{array}\right]$, $A_{22}=\left[\begin{array}{cc} 3.0612 & -0.8367\\ -0.5510 & 2.5306 \end{array}\right]$, ${\tilde{A}}_{21}=\left[\begin{array}{c} 1.1660\\ 1.0246 \end{array}\right]$, ${\tilde{A}}_{22}=\left[\begin{array}{cc} 3.0565 & -0.7839\\ -0.4982 & 1.9435 \end{array}\right]$, $\Phi =\left[\begin{array}{ccc} 1.0000 & 0 & 0\\ 0.0107 & 1.0000 & 0\\ -0.1187 & 0 & 1.0000 \end{array}\right]$. According to the prescribed-time Controller (41), the desired convergence time is T=1 s. Compared with [26], Figure 1 and Figure 2 show evolutions of the system states for $x_{0}=\left(0.5,0.2,0.3\right)$. It is clearly shown that the convergence time of the fixed-time control cannot be precisely fixed, except before the given time $T_{max}=1$ s. However, in the prescribed-time control process, the system states are stable just at the settling time T=1 s. Figure 3 and Figure 4 present the plots of the control magnitude. It is proven that the control magnitude is bounded by the prescribed-time strategy in Figure 3, but the excessive control gain by the fixed-time controller may exist while choosing the control parameters.*

**Example** **2.** *The model parameters of UUVs are adapted as follows in [27] and [28]: $m_{11}=200\;kg$, $m_{22}=250\;kg$, $m_{33}=80\;kg$, $d_{11}=\left(70+100|\rho |\right)\;kg/s$, $d_{22}=\left(100+200|v|\right)\;kg/s$, $d_{33}=\left(50+100|\vartheta |\right)\;kg/s$. There are three vehicles which are initialized as follows: $\left(x_{1},y_{1}\right)=\left(5\;m,5\;m\right)$, $\left(x_{2},y_{2}\right)=\left(5\;m,-5\;m\right)$, $\left(x_{3},y_{3}\right)=\left(1\;m,2\;m\right)$, $\left(x_{4},y_{4}\right)=\left(5\;m,1\;m\right)$, $\left(x_{5},y_{5}\right)=\left(-2\;m,3\;m\right)$, $\left(x_{6},y_{6}\right)=\left(2\;m,3\;m\right)$,$\;\rho_{1}=v_{1}=\rho_{2}=v_{2}=\rho_{3}=v_{3}=\rho_{4}=v_{4}=\rho_{5}=v_{5}=\rho_{6}=v_{6}=0\;m/s$, $\psi_{1}=\psi_{2}=\psi_{3}=\psi_{4}=\psi_{5}=\psi_{6}=0.1\;rad,\vartheta_{1}=\vartheta_{2}=\vartheta_{3}=\vartheta_{4}=\vartheta_{5}=\vartheta_{6}=0.1\;rad/s$. $\left(x_{rx1},x_{ry1}\right)=\left(5\;m,5\;m\right)$,$\left(x_{rx2},x_{ry2}\right)=\left(-5\;m,2\;m\right)$, $\left(x_{rx3},x_{ry3}\right)=\left(2\;m,1\;m\right)$. The prescribed-time is T=3s. The matrices $L_{1}=\left[3\;0\;0\;-1\;-1\;-1;\;-1\;1\;0\;0\;0\;0;\;-1\;-1\;2\;0\;0\;0;\;-1\;0\;0\;2\;0\;0;\;0\;0\;0\;-1\;2\;0;\;0\;0\;0\;0\;-1\;2\right],L_{2}=\left[0\;0\;0;\;0\;0\;0;\;0\;0\;0;\;0\;0\;-1;\;0\;-1\;0;\;-1\;0\;0\right]$.*

**Figure 5:**
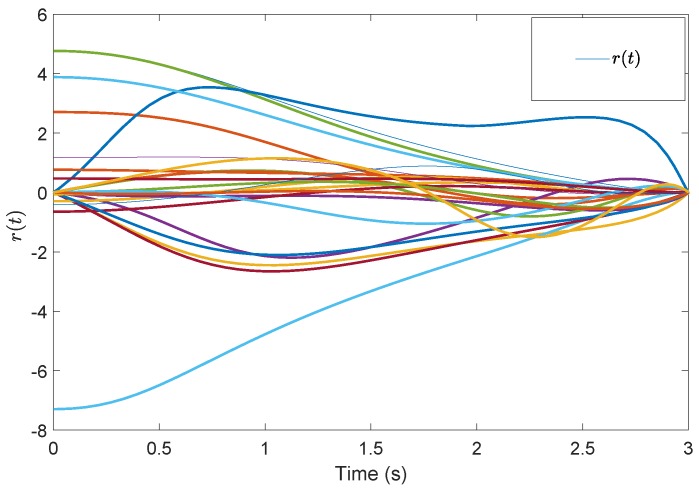
Containment consensus variable *r*(t) by the prescribed-time Controller (62).

**Figure 6:**
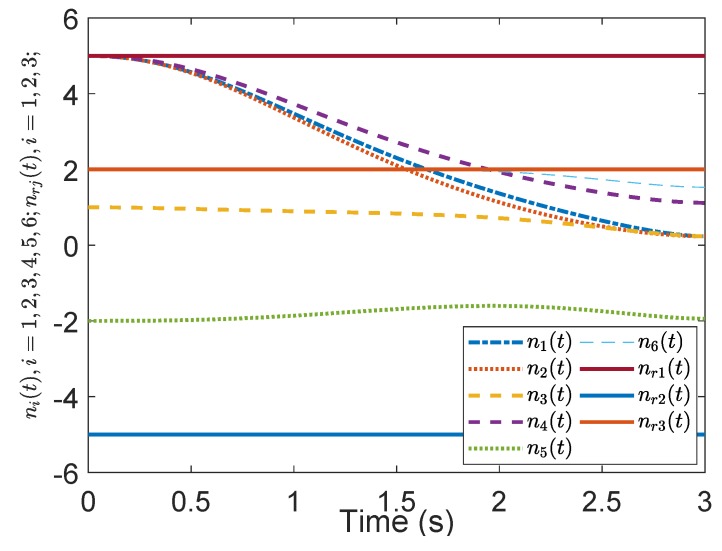
Position states *n*(t) by the prescribed-time Controller (62).

**Figure 7:**
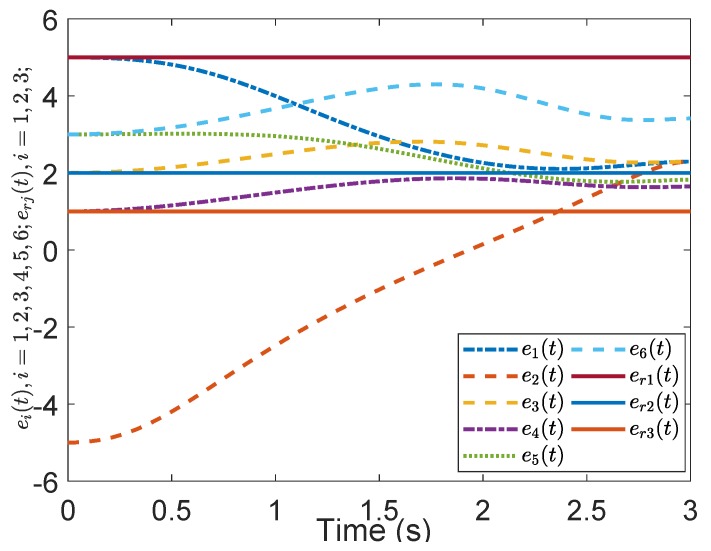
Position states *e*(t) by the prescribed-time Controller (62).

**Figure 8:**
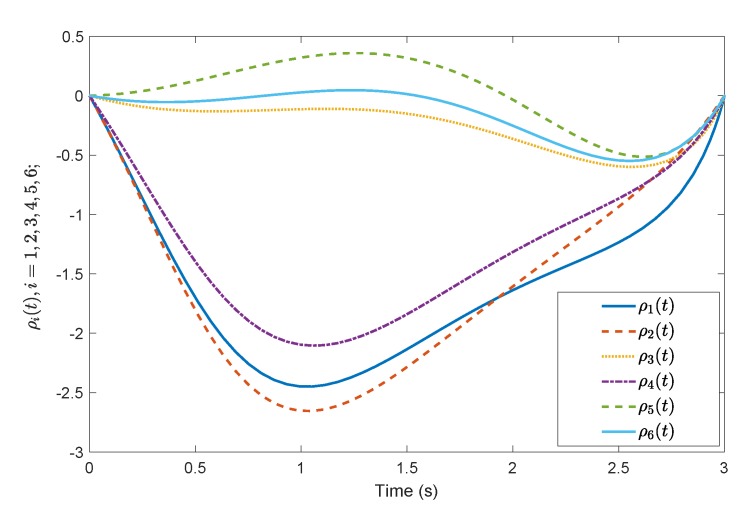
Velocity states ρ(t) by the prescribed-time Controller (62).

**Figure 9:**
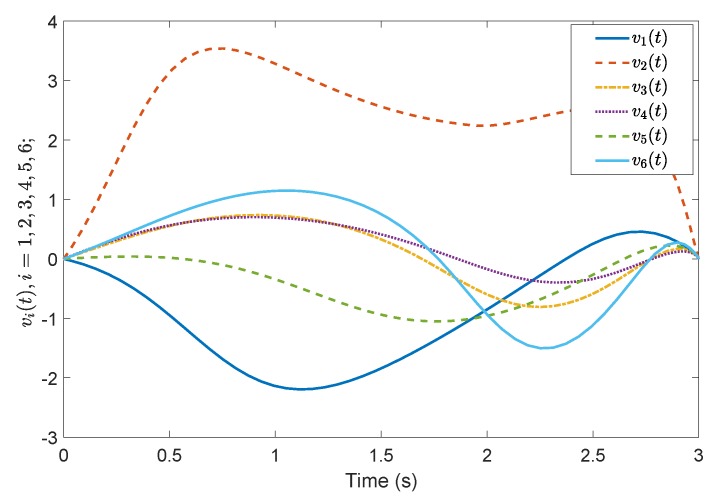
Velocity states *v*(t) by the prescribed-time Controller (62).

**Figure 10:**
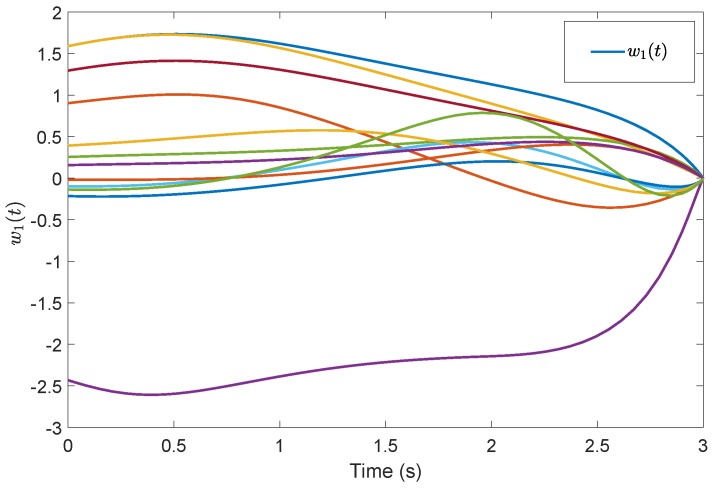
Intermediate states $w_{1}$(t) by the prescribed-time Controller (62).

**Figure 11:**
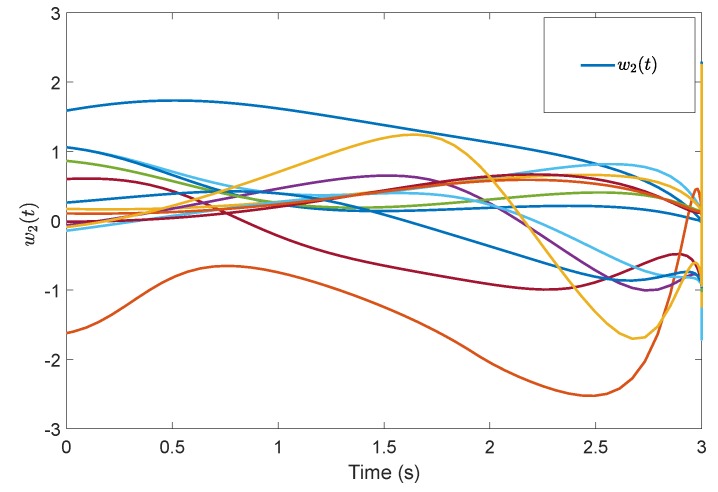
Intermediate states $w_{2}$(t) by the prescribed-time Controller (62).

**Figure 12:**
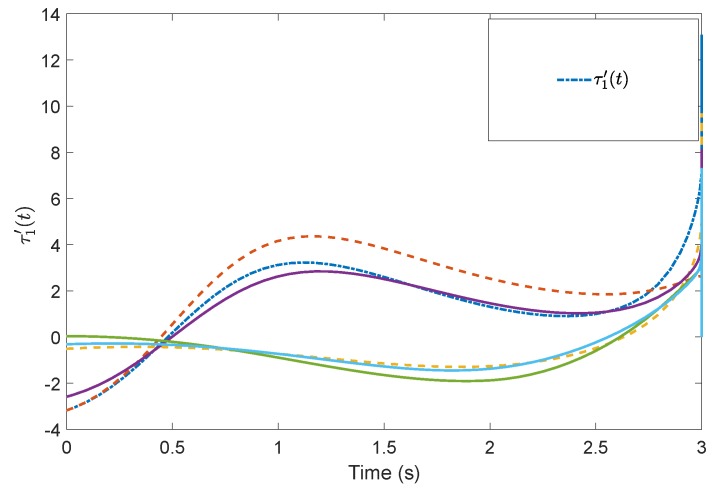
Control input $\tau_{1}^{\prime }$(t) by the prescribed-time Controller (62).

**Figure 13:**
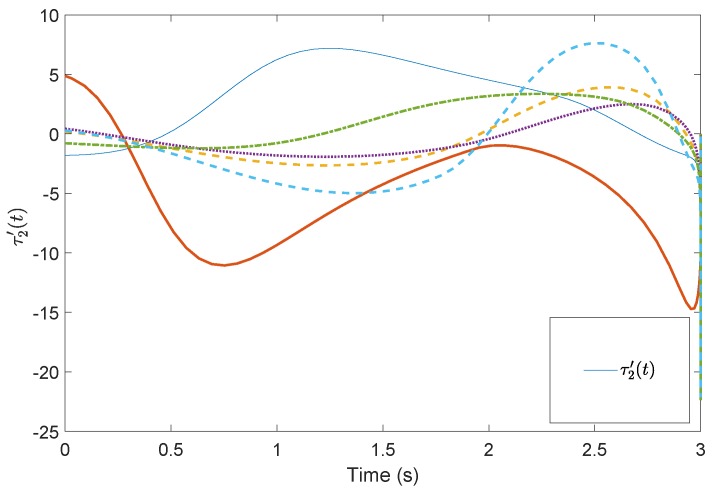
Control input $\tau_{2}^{\prime }$(t) by the prescribed-time Controller (62).

**Figure 14:**
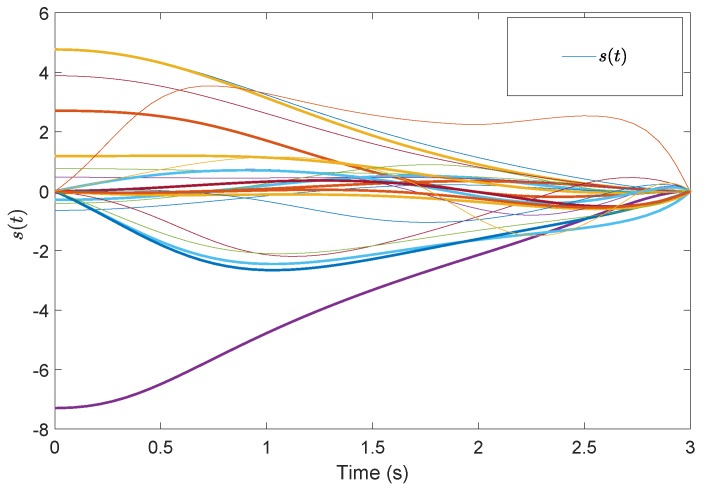
Intermediate states *s*(t) by the prescribed-time Controller (62).

Figure 5 shows the evolution of the containment consensus variable r(t) for the multiple UUVs converge to zero in the prescribed-time T=3 s. Figure 6 and Figure 7 are the standard position variables of UUVs in north and east, it shows that the position variables of the followers $n_{1},\cdots ,n_{6},i=1,\cdots ,6$ and $e_{1},\cdots ,e_{6},i=1,\cdots ,6$ converge into the convex hull formed by the leaders’ positions $n_{r1},n_{r2},n_{r3}$ and $e_{r1},e_{r2},e_{r3}$, respectively. Figure 8 and Figure 9 are the standard velocity variables of UUVs in surge and sway; they show that the velocity variables of the followers $\rho_{1},\cdots ,\rho_{6},i=1,\cdots ,6$ and $v_{1},\cdots ,v_{6},i=1,\cdots ,6$ converge to zero in the prescribed-time T=3 s. Figure 10, Figure 11, Figure 12, Figure 13 and Figure 14 show the trajectories of intermediate variables and the control inputs. Figure 10 shows that the variable $w_{1}$ converges to zero in the prescribed-time T=3 s. Figure 11 shows that the variable $w_{2}$ is prescribed-time uniformly bounded, which proves that the intermediate variable *w* is bounded. In Figure 12 and Figure 13, the control input $\tau_{1}^{\prime }$ and $\tau_{2}^{\prime }$ are shown to be prescribed-time uniformly bounded. In Figure 14, the intermediate state s(t) which is equivalent to r(t) converges to zero in the prescribed time. The effectiveness of the proposed prescribed-time controller is demonstrated.

To make the experimental results more comparative, consider the prescribe time as T=8 s. Then the validity of the proposed fixed-time observer is verified and the magnitudes of intermediate variables and containment control states can be effectively reduced in the following figures. The fixed time constant τ=1.5 s, and matrix $F_{a}=\left[0,\;1\right]$, the existing actuator faults are given below,

(63) $$\begin{array}{c} \begin{array}{c} f_{a1}=0.50\times \left(1.2-exp\left(-0.03t\right)\right)\\ f_{a2}=0.15\times \left(3.2-exp\left(-0.1t\right)\right)\\ f_{a3}=0.25\times \left(1.5-exp\left(-0.02t\right)\right)\\ f_{a4}=0.25\times \left(1.1-exp\left(-0.015t\right)\right)\\ f_{a5}=0.50\times \left(2.3-exp\left(-0.01t\right)\right)\\ f_{a6}=0.35\times \left(1.7-exp\left(-0.1t\right)\right) \end{array} \end{array}$$

**Figure 15:**
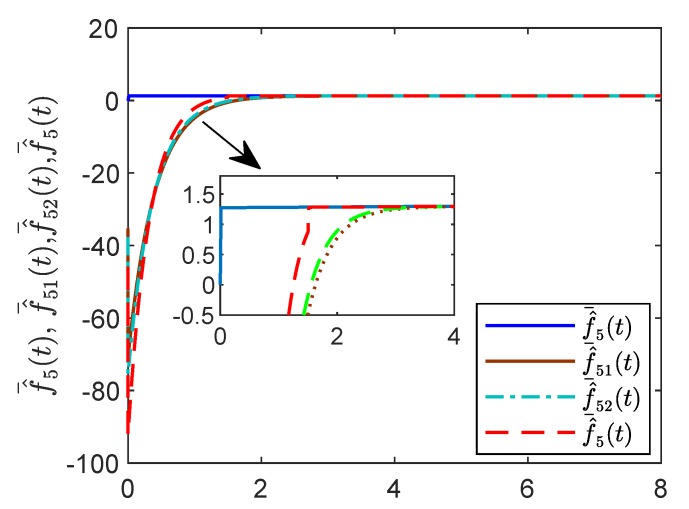
States $\hat{\bar{f}}$(t), ${\hat{\bar{f}}}_{51}$(t),${\hat{\bar{f}}}_{52}$(t),${\hat{\bar{f}}}_{5}$(t) by the fixed-time reduced-order controller.

**Figure 16:**
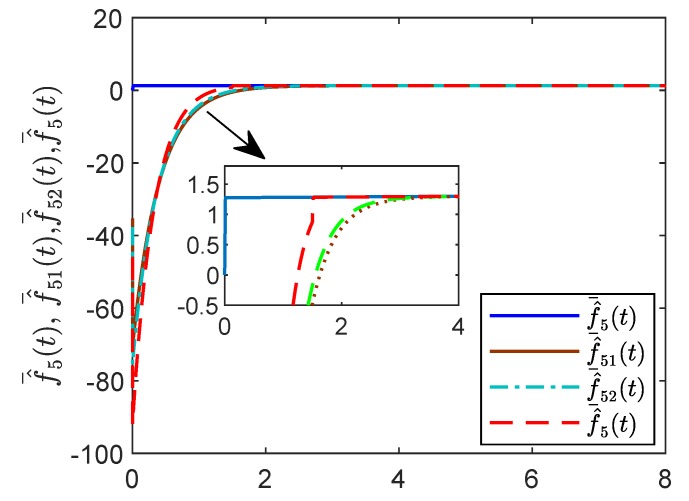
States ‖r‖ with and without observer.

**Figure 17:**
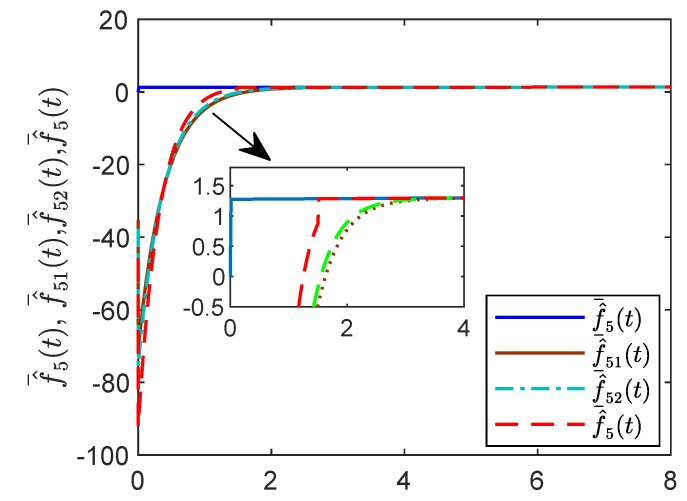
States ‖w‖ with and without observer.

For reasons of length and simplicity, only the estimation process of ${\bar{f}}_{5}\left(t\right)$ is shown. The trajectories of states ${\bar{f}}_{5}\left(t\right)$, ${\hat{\bar{f}}}_{51}\left(t\right)$, ${\hat{\bar{f}}}_{52}\left(t\right)$ and ${\hat{\bar{f}}}_{5}\left(t\right)$ are given in Figure 15. It is shown that ${\bar{f}}_{5}\left(t\right)$ can be estimated by ${\hat{\bar{f}}}_{5}\left(t\right)$ at time t=1.5 s, while ${\hat{\bar{f}}}_{51}\left(t\right)$ and ${\hat{\bar{f}}}_{52}\left(t\right)$ can estimate ${\bar{f}}_{5}\left(t\right)$ as time goes to infinity. In Figure 16 and Figure 17, the effect of the observer on the containment variable *r* and intermediate variable *w* are given. When t>τ the fixed-time observer can work and effectively reduce the magnitude of the containment variable r(t) and the intermediate variable w(t). The effectiveness of the proposed fixed-time algorithm is proved.

## 5. Conclusions

The paper presents the prescribed-time containment consensus control for multiple UUV systems with nonlinear uncertainties and disturbances. The control design procedures utilize the block decomposition technique and Lyapunov control theorem. This approach allows us to converge the containment consensus variable in the prescribed time. In addition, intermediate variables and control input are also shown to remain uniformly bounded. To reduce the magnitude of the bounds, a novel fixed-time observer for the faults is proposed. Due to the fact that the sensor fault may exist, and the event-triggered mechanism can reduce the burden of communication that may be interesting and meaningful for the complex ocean environment; both of these will be chosen as our future directions.

## Appendix A. Proof of Lemma 3

We can rewrite the position and orientation transforms described in System (3) as [29]

(A1) $$\begin{array}{ll} {\ddot{p}}_{i} & =\dot{R}\left(\psi_{i}\right)\epsilon_{i}+R\left(\psi_{i}\right){\dot{\epsilon }}_{i}=R\left(\psi_{i}\right)S\left(\vartheta_{i}\right)\epsilon_{i}+R\left(\psi_{i}\right){\dot{\epsilon }}_{i}\\ & =R\left(\psi_{i}\right)[S\left(\vartheta_{i}\right)-M_{1}^{-1}P(\vartheta_{i},|\varrho_{i}\left|,\right|v_{i}\left|)\right]R^{-1}\left(\psi_{i}\right){\dot{p}}_{i}+R\left(\psi_{i}\right)M_{1}^{-1}\tau_{i}\\ & =G(\psi_{i},\vartheta_{i},|\varrho_{i}\left|,\right|v_{i}\left|\right){\dot{p}}_{i}+H\left(\psi_{i}\right)\tau_{i} \end{array}$$

with $G(\psi_{i},\vartheta_{i},|\varrho_{i}\left|,\right|v_{i}\left|\right)=R\left(\psi_{i}\right)[S\left(\vartheta_{i}\right)-M_{1}^{-1}P(\vartheta_{i},|\rho_{i}\left|,\right|v_{i}\left|)\right]R^{-1}\left(\psi_{i}\right)$, $S\left(\rho_{i}\right)=\left[\begin{array}{cc} 0 & -\rho_{i}\\ \rho_{i} & 0 \end{array}\right]$, $M_{1}=\left[\begin{array}{cc} m_{11} & 0\\ 0 & m_{22} \end{array}\right]$, $P(\vartheta_{i},|\varrho_{i}\left|,\right|v_{i}\left|\right)=\left[\begin{array}{cc} d_{11} & -m_{22}\vartheta_{i}\\ m_{11}\vartheta_{i} & d_{22} \end{array}\right]$. With $x_{fi}={\left[p_{i}^{T},\epsilon_{i}^{T}\right]}^{T}$, we can easily obtain Dynamic (4). The proof is completed. For more details see [29].

## Appendix B. Proof of Theorem 1

Let $e_{i}\left(t\right)={\left[{\left({\hat{\bar{f}}}_{i1}\left(t\right)-{\bar{f}}_{i}\left(t\right)\right)}^{T}\;{\left({\hat{\bar{f}}}_{i2}\left(t\right)-{\bar{f}}_{i}\left(t\right)\right)}^{T}\right]}^{T}$; the estimation error dynamics ${\dot{e}}_{i}$ can be obtained

(A2) $$\begin{array}{c} {\dot{e}}_{i}\left(t\right)=\left[\begin{array}{cc} M_{i1} & 0\\ 0 & M_{i2} \end{array}\right]e_{i}\left(t\right)-\Delta_{i}\left(t\right) \end{array}$$

with $\Delta_{i}=\left[\begin{array}{c} T_{i}\bar{D}\\ T_{i}\bar{D} \end{array}\right]{\bar{\beta }}_{i}$. Then

(A3) $$\begin{array}{c} e_{i}\left(t-\tau \right)=e^{-{\bar{M}}_{i}\tau }e_{i}\left(t\right)-\int_{t-\tau }^{t}e^{{\bar{M}}_{i}\left(t-s-\tau \right)}\Delta_{i}\left(s\right)ds \end{array}$$

Then when t>τ we have

(A4) $$\begin{array}{ll} {\hat{\bar{f}}}_{i}\left(t\right) & =K_{i}\left(\rho_{i}\left(t\right)-e^{{\bar{M}}_{i}\tau }\rho_{i}\left(t-\tau \right)\right)\\ & ={\bar{f}}_{i}\left(t\right)+\left[I\;\;0\right]{\left[\begin{array}{cc} I & e^{{\bar{M}}_{i1}\tau }\\ I & e^{{\bar{M}}_{i2}\tau } \end{array}\right]}^{-1}\left(\rho_{i}\left(t\right)-1{\bar{f}}_{i}\left(t\right)-e^{{\bar{M}}_{i}\tau }\left(\rho_{i}\left(t-\tau \right)-1{\bar{f}}_{i}\left(t-\tau \right)\right)\right)\\ & ={\bar{f}}_{i}\left(t\right)+\left[I\;\;0\right]{\left[\begin{array}{cc} I & e^{{\bar{M}}_{i1}\tau }\\ I & e^{{\bar{M}}_{i2}\tau } \end{array}\right]}^{-1}\left(e_{i}\left(t\right)-e^{{\bar{M}}_{i}\tau }e_{i}\left(t-\tau \right)\right)\\ & ={\bar{f}}_{i}\left(t\right)+\left[I\;\;0\right]{\left[\begin{array}{cc} I & e^{{\bar{M}}_{i1}\tau }\\ I & e^{{\bar{M}}_{i2}\tau } \end{array}\right]}^{-1}\int_{t-\tau }^{t}e^{{\bar{M}}_{i}\left(t-s\right)}\Delta_{i}\left(s\right)ds \end{array}$$

with the fact K1=I and $Ke^{{\bar{M}}_{i}\tau }1=0$. Due to the fact that $\Delta_{i}\left(s\right)$ is bounded, we known the last integral term is bounded. Then we can find a constant α such that $|{\hat{\bar{f}}}_{i}\left(t\right)-{\bar{f}}_{i}\left(t\right)|<\alpha$.

Further, the existence of matrices $M_{i}$, $G_{i}$, $R_{i}$ satisfying Conditions (18) can be found in [30], which is omitted here. The proof is completed.

## Appendix C. Proof of Lemma 4

Due to the fact that the desired transformed system is

(A5) $$\begin{array}{c} {\dot{w}}_{i}=\frac{-k_{1}}{T-t}w_{i}+A_{i,i+1}w_{i+1} \end{array}$$

to guarantee the coefficient of $a_{\mathrm{ii}}$ will be an identity matrix, we design the feasible solution as follows,

(A6) $$\begin{array}{c} w_{i+1}=A_{i,i+1}^{+}\left({\dot{w}}_{i}+\frac{k_{1}}{T-t}w_{i}\right)+\left(I-A_{i,i+1}^{+}A_{i,i+1}\right)\frac{s_{i+1}}{T-t} \end{array}$$

In fact, the solutions to $w_{i+1}$ are infinite, the choice of $\left(I-A_{i,i+1}^{+}A_{i,i+1}\right)\frac{s_{i+1}}{T-t}$ results in the coefficient of controller *u* being $A_{k,k+1}^{+}$, which is row full rank.

According to the general form of $w_{i}$, the derivative of $w_{i}$ is obtained as

(A7) $$\begin{array}{c} {\dot{w}}_{i}=\frac{s_{i}}{{\left(T-t\right)}^{2}}+\frac{A_{i,i+1}s_{i+1}}{T-t}+\sum_{j=1}^{i-1}\frac{a_{i,j}\left(i+1-j\right)}{{\left(T-t\right)}^{i+2-j}}s_{j}+\sum_{j=1}^{i-1}\frac{a_{i,j}A_{j,\;j+1}}{{\left(T-t\right)}^{i+1-j}}s_{j+1} \end{array}$$

Put Equation (A7) into Equation (A6), then one has

(A8) $$\begin{array}{ll} w_{i+1} & =A_{i,i+1}^{+}(\frac{s_{i}}{{\left(T-t\right)}^{2}}+\frac{A_{i,i+1}s_{i+1}}{T-t}+\sum_{j=1}^{i-1}\frac{a_{i,j}\left(i+1-j\right)}{{\left(T-t\right)}^{i+2-j}}s_{j}+\sum_{j=1}^{i-1}\frac{a_{i,j}A_{j,\;j+1}}{{\left(T-t\right)}^{i+1-j}}s_{j+1}\\ & +\frac{k_{1}}{T-t}\left(\frac{s_{i}}{T-t}+\sum_{j=1}^{i}\frac{a_{i,j}}{{\left(T-t\right)}^{i+1-j}}s_{j}\right))+\left(I-A_{i,i+1}^{+}A_{i,i+1}\right)\frac{s_{i+1}}{T-t} \end{array}$$

Thus,

(A9) $$\begin{array}{ll} w_{i+1} & =A_{i,i+1}^{+}(\frac{s_{i}}{{\left(T-t\right)}^{2}}+\frac{A_{i,i+1}s_{i+1}}{T-t}+\sum_{j=1}^{i-1}\frac{a_{i,j}\left(i+1-j\right)}{{\left(T-t\right)}^{i+2-j}}s_{j}+\sum_{j=1}^{i-1}\frac{a_{i,j}A_{j,\;j+1}}{{\left(T-t\right)}^{i+1-j}}s_{j+1}+\frac{{k_{1}s}_{i}}{{\left(T-t\right)}^{2}}\\ & +\sum_{j=1}^{i}\frac{{k_{1}a}_{i,j}}{{\left(T-t\right)}^{i+2-j}}s_{j})+\left(I-A_{i,i+1}^{+}A_{i,i+1}\right)\frac{s_{i+1}}{T-t}\\ & =\frac{s_{i+1}}{T-t}+A_{i,i+1}^{+}\left(\frac{\left(k+1\right)s_{i}}{{\left(T-t\right)}^{2}}+\sum_{j=1}^{i-1}\frac{a_{i,j}\left(i+1-j+k_{1}\right)}{{\left(T-t\right)}^{i+2-j}}s_{j}+\sum_{j=1}^{i-1}\frac{a_{i,j}A_{j,\;j+1}}{{\left(T-t\right)}^{i+1-j}}s_{j+1}\right)\\ & =\frac{s_{i+1}}{T-t}+A_{i,i+1}^{+}\left(\sum_{j=1}^{i}\frac{a_{i,j}\left(i+1-j+k_{1}\right)}{{\left(T-t\right)}^{i+2-j}}s_{j}+\sum_{j=1}^{i-1}\frac{a_{i,j}A_{j,\;j+1}}{{\left(T-t\right)}^{i+1-j}}s_{j+1}\right) \end{array}$$

Extracting the coefficients of the term $s_{j}$ from $\sum_{j=1}^{i-1}\frac{a_{i,j}A_{j,\;j+1}}{{\left(T-t\right)}^{i+1-j}}s_{j+1}$, then we have

$$w_{i+1}=\frac{s_{i+1}}{T-t}+A_{i,i+1}^{+}\left(\sum_{j=1}^{i}\frac{a_{i,j}\left(i+1-j+k_{1}\right)}{{\left(T-t\right)}^{i+2-j}}s_{j}+\sum_{j=1}^{i}\frac{a_{i,j-1}A_{j-1,\;j}}{{\left(T-t\right)}^{i+2-j}}s_{j}\right)$$

Then we have

(A10) $$\begin{array}{c} w_{i+1}=\frac{s_{i+1}}{T-t}+A_{i,i+1}^{+}\left(\sum_{j=1}^{i}\frac{a_{i,j}\left(i+1-j+k_{1}\right)+a_{i,j-1}A_{j-1,\;j}}{{\left(T-t\right)}^{i+2-j}}s_{j}\right) \end{array}$$

To satisfy Condition (28), we obtain

(A11) $$\begin{array}{c} a_{i+1,j}=A_{i,i+1}^{+}\left(a_{i,j}\left(i+1-j+k_{1}\right)+a_{i,j-1}A_{i-1,j}\right) \end{array}$$

The proof is completed.

## Appendix D. Proof of Lemma 5

Take the derivative of Equation (34) on both sides,

(A12) $$\begin{array}{ll} A_{i,i+1}s_{i+1} & =\sum_{j=1}^{i-1}-b_{i,j}\left(1-i+j\right){\left(T-t\right)}^{-i+j}w_{j}+\sum_{j=1}^{i-1}b_{i,j}{\left(T-t\right)}^{1-i+j}\left(-\frac{k_{1}}{T-t}w_{j}+A_{j,j+1}w_{j+1}\right)\\ & -\left(k_{1}+1\right)w_{i}+A_{i,i+1}w_{i+1}\left(T-t\right)\\ & =\sum_{j=1}^{i-1}-b_{i,j}\left(1-i+j+k_{1}\right){\left(T-t\right)}^{-i+j}w_{j}+\sum_{j=1}^{i-1}b_{i,j}{\left(T-t\right)}^{1-i+j}A_{j,j+1}w_{j+1}-\left(k_{1}+1\right)w_{i}\\ & +A_{i,i+1}w_{i+1}\left(T-t\right)\\ & =\sum_{j=1}^{i}{-b}_{i,j}\left(1-i+j+k_{1}\right){\left(T-t\right)}^{-i+j}w_{j}+b_{i,i}\left(1+k_{1}\right)w_{i}+\sum_{j=1}^{i-1}b_{i,j}{\left(T-t\right)}^{1-i+j}A_{j,j+1}w_{j+1}\\ & -\left(k_{1}+1\right)w_{i}+A_{i,i+1}w_{i+1}\left(T-t\right)\\ & =\sum_{j=1}^{i}-b_{i,j}\left(1-i+j+k_{1}\right){\left(T-t\right)}^{-i+j}w_{j}+\sum_{j=1}^{i}b_{i,j-1}{\left(T-t\right)}^{-i+j}A_{j-1,j}w_{j}+A_{i,i+1}w_{i+1}\left(T-t\right) \end{array}$$

with $b_{i,q}=0$, $b_{i,0}=0.$ Then

(A13) $$\begin{array}{ll} s_{i+1} & =A_{i,i+1}^{+}\sum_{j=1}^{i+1}\left({-b}_{i,j}\left(1-i+j+k_{1}\right)+b_{i,j-1}A_{j-1,j}\right){\left(T-t\right)}^{-i+j}w_{j}\\ & +A_{i,i+1}^{+}A_{i,i+1}w_{i+1}\left(T-t\right)+\left(I-A_{i,i+1}^{+}A_{i,i+1}\right)w_{i+1}\left(T-t\right)\\ & =A_{i,i+1}^{+}\sum_{j=1}^{i+1}\left({-b}_{i,j}\left(1-i+j+k_{1}\right)+b_{i,j-1}A_{j-1,j}\right){\left(T-t\right)}^{-i+j}w_{j}+\left(T-t\right)w_{i+1} \end{array}$$

One has

(A14) $$\begin{array}{c} b_{i+1,j}=A_{i,i+1}^{+}\left(-b_{i,j}\left(1-i+j+k_{1}\right)+b_{i,j-1}A_{j-1,j}\right) \end{array}$$

Thus

$$s_{i+1}=\sum_{j=1}^{i}b_{i+1,j}{\left(T-t\right)}^{-i+j}w_{j}+\left(T-t\right)w_{i+1}$$

The proof is completed.
